# Association study of single nucleotide polymorphism in tryptophan hydroxylase 1 gene with adolescent idiopathic scoliosis: A meta-analysis

**DOI:** 10.1097/MD.0000000000023733

**Published:** 2021-01-22

**Authors:** Junyu Li, Zexi Yang, Miao Yu

**Affiliations:** a Orthopedic Department, Peking University Third Hospital, 49 North Garden Road; b School of Clinical Medicine, Peking University Health Science Center, 38 Xueyuan Road, Haidian District, Beijing 100191, China.

**Keywords:** adolescent idiopathic scoliosis, genetic association study, meta-analysis, single nucleotide polymorphism, TPH1

## Abstract

**Background:**

Adolescent idiopathic scoliosis is a common spinal deformity among children and adolescents worldwide with its etiology uncertain. Over a decade, a single nucleotide polymorphism rs10488682 in tryptophan hydroxylase 1 (TPH1) gene has been investigated in several association studies. We perform this study to summarize the current evidence of TPH1 rs10488682 polymorphisms and adolescent idiopathic scoliosis (AIS).

**Methods:**

Six databases were systematically searched: PubMed, Embase, Cochrane Library, Web of Science, Chinese Biomedical Literature, and Wanfang database. Eligible case–control studies related to TPH1 and AIS were selected. Reference lists of them were reviewed for more available studies. Two authors independently screened and evaluated the literature and extracted data. The odds ratios and 95% confidence intervals were derived in association tests. Subgroup analysis was conducted by ethnicity. Sensitivity analysis was performed to examine the stability of the overall results.

**Results:**

A total of 1006 cases and 1557 controls in 3 independent studies were included for meta-analysis. Statistical significance was discovered in heterozygote model (AT vs AA: OR = 1.741, 95%Cl = 1.100–2.753, *P* = .018 < .05, *I*^2^ = 0%), recessive model (AA vs AT + TT: OR = 0.640, 95%Cl = 0.414–0.990, *P* = .045 < .05, *I*^2^ = 0%) and over-dominant model (AT vs AA + TT: OR = 1.366, 95%Cl = 1.115–1.673, *P* = .003 < .05, *I*^2^ = 84.7%) in overall populations. Similar associations were also found in the Caucasian population. No significant associations were found in other genotypic comparisons and allelic comparisons.

**Conclusions:**

Statistically significant correlations were discovered between the TPH1 rs10488682 polymorphisms and AIS. Heterozygous AT genotype seems to be risky with an over-dominant effect. Ethnicity appears to modify the disease association.

**Registration:**

Not applicable.

## Abstract 2

Supplemental Digital Content is available in the text

## 1 Introduction

Adolescent idiopathic scoliosis (AIS) is a common spinal deformity among children near the onset of puberty worldwide. Scoliosis is term to describe a lateral curvature of the spine and is often accompanied by varying degrees of spinal rotation.^[1]^ Between 1% and 4% of adolescents are affected in the early puberty with rapid growth until sexual maturity. Females are more susceptible in most of the cases.^[2]^ It is well known for its rapid curve development and it has a progressive trend during the growth of a child.^[3]^ Cobb angle is widely accepted to describe the severity of the disease with a cut-off point at 10 degrees.^[4]^

Researchers have been looking into the pathogenesis of idiopathic scoliosis for decades, but the answers are not confirmed. Possible cause of AIS includes abnormal structure of paraspinal muscle and connective tissue, secretion of growth hormone, circadian rhythm, and so on.^[5,6]^ Studies have shown that approximately 25% of AIS patients have a family history. Also, twin studies have endorsed the role of genetic factors.^[7,8]^ With the widespread of PCR technology in clinic, genetic researches, including linkage studies, association studies and genome-wide association studies (GWAS), have become a hot spot in the past decade. Several possible predisposition or disease-modifier genes have been reported.^[3]^ It has been reviewed that candidate genes of AIS consisted of ones that related to connective tissue structure, bone formation and metabolism, melatonin pathway, and puberty and growth.^[9]^

Up to now, several gene loci have been investigated or even meta-analyzed. It is not rare to see one study reports a significant association while the following ones refute the previous hypothesis. This phenomenon of “false-positive first report” may due to demographic characteristics and research design. Vitamin D receptor (VDR) gene, which is related to bone formation, has been reported a significant association with AIS at locus Bsml rs1544410 and Apal rs7975232.^[10]^ Meanwhile, another review only approved the result in Bsml but rejected the conclusion in Apal.^[11]^ Estrogen receptor (ESR) gene is believed to be related to growth and maturation. Nevertheless, null association with AIS was found in the review of both locus ESR1 rs9340799 and ESR2 rs1256120.^[12,13]^ In addition, investigation of insulin-like growth factor gene at locus IGF1 rs5742612 yielded no correlation.^[14]^ However, positive result was suggested in ladybird homeobox gene at locus LBX1 rs11190870, which was confirmed by 3 reviews published in different years.^[15–17]^

Apart from these, genes related to the melatonin metabolic pathways, such as MTNR1,^[18,19]^ tryptophan hydroxylase 1 (TPH1),^[20]^ AANAT,^[21]^ have drawn great attention. Melatonin is primarily synthesized by pineal gland, which is implicated in the control of circadian rhythms and also believed to cause the occurrence of idiopathic scoliosis.^[22]^ Prior experiments showed that chickens hardly had any melatonin left after pinealectomy and then developed spinal curvature. But implantation of the pineal gland in muscle prevented the development of experimentally induced scoliosis.^[23,24]^ Thus, melatonin pathway is a vital mechanism that might lead to the onset and progression of deformity.^[25]^

TPH1 gene has been investigated in several genetic association studies. In 2008, Hai Wang was the first author who investigated the association of TPH1 rs10488682 with AIS and got positive results.^[21]^ In 2011, Xu found positive results that TPH1 gene could significantly influence the effect of brace treatment in a case-only study.^[26]^ Over the past decade, many researchers from different countries have studied this gene locus from many perspectives including predisposition, progression, and treatment prognosis.^[20,26–28]^ However, they got controversial conclusions. In the cause of enhancing the statistical power of single studies, we performed this meta-analysis to review the studies and summarized the available evidence whether TPH1 rs10488682 polymorphisms are correlated with AIS.

In this study, we selected and analyzed case–control studies with reference to PRISMA checklist, which investigated the genetic distribution of TPH1 rs10488682 gene locus in AIS patients and compared with the healthy control groups.

## 2 Methods

### 2.1 Literature search

This study was performed according to the PRISMA guidelines. We systematically searched all the common databases until February 2020, including: PubMed, Embase, Cochrane Library, Web of Science, Chinese Biomedical Literature, and Wanfang database. A combination of terms was used: TPH1, tryptophan hydroxylase 1; AIS, adolescent idiopathic scoliosis; SNP, single nucleotide polymorphism; case–control study. We especially featured in the genetic predisposition of AIS, with regard to the association between TPH1 gene and AIS disease. There was no language restriction. Secondary screening of literature was performed through reference lists of selected articles. Details of search method are presented in the supplementary materials.

### 2.2 Eligible studies criteria

We screened all of the searching results and evaluated full-text reports for potentially eligible studies. Two reviewers independently screened and evaluated the literature. A third reviewer solved the contradictions. Studies were included if they met following items:

(1) case–control study;
(2) investigation of target gene TPH1 rs10488682 polymorphisms of all samples;
(3) clinical diagnosis of AIS based on medical and/or radiological examination;
(4) sufficient and accurate data, including sample count or frequency of alleles and genotypes, or both.

Studies were excluded if:

(1) case report, clinical trial, or review;
(2) no specific data of genetic distribution of all samples;
(3) duplicated studies.

### 2.3 Quality assessment

Two reviewers independently evaluated the methodological quality of studies by the Newcastle-Ottawa Scale (NOS) for case–control studies. Articles scored 5 stars or more among the all 9 items were considered to have enough methodological quality and thus included in our study. The Hardy–Weinberg equilibrium (HWE) of all study controls was also assessed. Publication and selective bias were evaluated by funnel plots.

### 2.4 Data extraction

Two authors independently collected useful data from eligible studies. In case of confusing or missing part of the literature, both authors managed to reach a consensus by discussing or consulting a senior author. Following contents (if included) were extracted: authors, country and region, publication date, sex, ethnicity, case selection criteria, sampling method and source, blinding, sample size, genotyping method, allele and genotype frequencies, genetic equilibrium, and population stratification.

### 2.5 Statistical analysis

Our meta-analysis was performed by STATA 15.1 SE (StataCorp, Texas 77845). We did not restrict any possible genetic model in advance. Comparisons of summary odds ratios and 95% confidence intervals were estimated to assess the link between the TPH1 rs10488682 polymorphisms and AIS. The Mantel–Haenszel analysis was used to calculate summary ORs of the allele comparison (A vs T), codominant heterozygote model (AT vs TT and AT vs AA), homozygote model (AA vs TT), dominant model (AA + AT vs TT), recessive model (AA vs AT + TT), and over-dominant model (AT vs AA + TT), where a *Z* test *P* < .05 indicated statistical significance. Each genetic model was examined to fit the most appropriate models. Heterogeneity between each study was detected by the Chi-squared-based *Q* test and quantified by the *I*^2^ value. Random-effect model was used in case of observable heterogeneity when *I*^2^ > 50% or a *P* < .10. Fixed-effect model was used otherwise. Because of possible ethnic differences in AIS, subgroup analysis by ethnicity was introduced to assess its impact on our results and whether it was a potential source of heterogeneity. Sensitivity analysis was used to assess the stability of summary results and find the source of heterogeneity.

## 3 Results

### 3.1 Study characteristics

**Figure 1:**
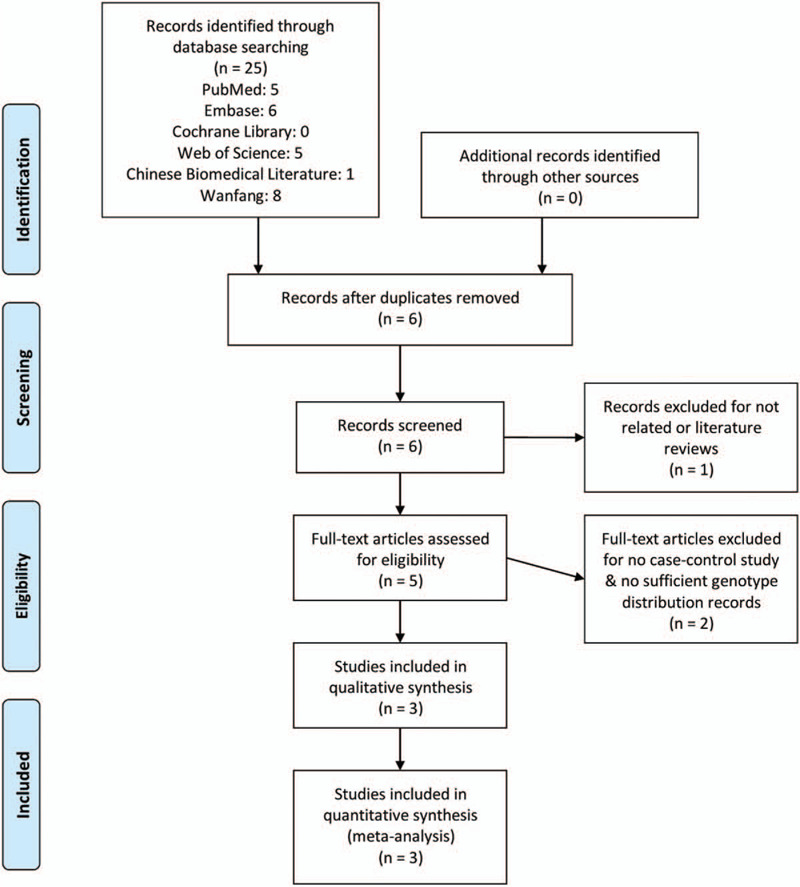
Flow diagram of the literature searching, identification, and selection process.

The literature searching, identification, and selection process (Fig. 1) yielded 3 studies that could be included in our meta-analysis.^[21,27,28]^ All of them were case–control studies of TPH1 rs10488682 polymorphisms and AIS. In the end, a total of 1006 cases and 1557 controls were qualified for analysis. Every article was published in English language. Population conducted in these studies consisted of Chinese, Japanese, and Bulgarian. The general literature information was summarized in Table 1 and the genetic characteristic (alleles and genotypes frequencies) was listed in Table 2.

**Table 1:** General characteristics of studies included in the meta-analysis.

No.	Study	Country	Ethnicity	Sex	Genotyping method	Case characteristics	Control characteristics	Subject selection criteria
1	Yablanski V^[28]^ (2016)	Bulgaria	Eastern European	Overall	PCR-RFLP, peripheral blood leucocytes	n = 105; with male/female = 19/86, and progressive/slowly progressive = 84/21; Cobb angle: progressive = 62.7 ± 17.4° and slowly progressive = 22.1 ± 6.3°); mean age at the beginning of the disease: 11.2 ± 3.1; age range: no report; Bulgarian.	n = 210; healthy subjects and adult patients with skeletal maturity without family history of IS; matched in gender and ethnicity.	Recruited by surgeons, diagnosis radiologically; divided by Cobb angle; excluded: secondary scoliosis, etc.
2	Wang H^[21]^ (2008)	China	Han Chinese	Overall	PCR-RFLP, 4 ml of blood	n = 103; Cobb angle: >30°; mean age: no report; age range: 10–20; Han Chinese.	n = 108; non-scoliosis subjects; matched in gender, age and ethnicity.	Diagnosis clinically and radiologically; excluded: congenital, neuromuscular, infantile, juvenile, secondary scoliosis, etc.
3	Takahashi Y^[27]^ (2011)	Japan	Japanese	Female	PCR-based invader assay, peripheral blood leucocytes	n = 798; all female; Cobb angle: >15°; mean age: 17.7 ± 5.8; age of diagnosis: 10–18; Japanese.	n = 1239; all female; patients without scoliosis and healthy subjects; mean age: 63.7 ± 13.8; Japanese.	Diagnosis by clinical and radiologic examinations; excluded: alternate diagnoses of congenital, juvenile, adult-onsets, secondary scoliosis, etc.

**Table 2:** Alleles and genotypes distributions of studies included in the meta-analysis.

			Case						Control					
			Allele		Genotype				Allele		Genotype			
Study	Patient group	Sample size	A	T	AA	AT	TT	Sample size	A	T	AA	AT	TT	HWE p
Yablanski V^[28]^ (2016)	Progressive	84	122	46	43	36	5	210	328	92	135	58	17	0.00522202
	None or slowly progressive	21	30	12	11	8	2							
Wang H^[21]^ (2008)	/	103	41	165	0	41	62	108	17	199	0	17	91	0.374656501
Takahashi Y^[27]^ (2011)	/	798	143	1453	5	133	660	1239	207	2271	8	191	1040	0.810577463

Total		1006	336	1676	59	218	729	1557	552	2562	143	266	1148	

### 3.2 Quality assessment

Hardy–Weinberg equilibrium test was conducted in the controls of these studies. Slight deviation from equilibrium was detected in the Bulgarian study by Yablanski. We found its *P* value .0052 < .05, which might indicate selection bias. In studies by Wang and Takahashi, genotypes were in Hardy–Weinberg equilibrium.

Besides, with the use of the NOS (Table 3) for case–control studies, we believed these studies have sufficient methodological quality to meet the criteria. The number of included studies was too small (n < 10) to detect publication bias and was not sufficient to explain the funnel plots.

**Table 3:** Quality assessment (using a modified Newcastle-Ottawa Scale) of studies included in the meta-analysis.

Study	Adequacy of case definition	Representativeness of the cases	Selection of controls	Definition of controls	Match of cases and controls	Population stratification	Ascertainment of exposure	Same method for cases and controls	Non-response rate	Total scores
Yablanski V^[28]^ (2016)	Independent validation with medical records and radiographs (a)	Cases from defined medical centers with outcome of interest over a defined period of time (a)	Same community as cases (a)	No history of AIS (a)	No description (c)	No description (c)	Secure record genotyping (a)	Yes (a)	Not reported (b)	6
Wang H^[21]^ (2008)	Independent validation with medical records and radiographs (a)	Cases from defined medical centers with outcome of interest over a defined period of time (a)	Hospitalized population (b)	No history of AIS (a)	Matched in gender and age (a)	No description (c)	Secure record genotyping (a)	Yes (a)	Not reported (b)	6
Takahashi Y^[27]^ (2011)	Independent validation with medical records and radiographs (a)	Cases from defined medical centers with outcome of interest over a defined period of time (a)	Both hospitalized and community population (b)	No history of AIS (a)	Matched in gender (a)	No description (c)	Secure record genotyping (a)	Yes (a)	Not reported (b)	6

### 3.3 Meta-analysis results

The results of summary effect and heterogeneity tests of rs10488682 polymorphisms of TPH1 gene and AIS are shown in Table 4.

**Table 4:** Results of genetic models for TPH1 rs10488682 polymorphisms and AIS.

		Test of association		Test of heterogeneity	
Comparison	N	OR (95%Cl)	*P*	Model	*I*^2^ (%)
Overall	3				
A vs T		1.251 (0.696–2.249)	.454	Random	86.1
AT vs AA		1.741 (1.100–2.753)	.018	Fixed	0
AT vs TT		1.855 (0.820–4.195)	.138	Random	82.6
AA vs TT		0.990 (0.488–2.007)	.977	Fixed	0
AA + AT vs TT		1.657 (0.756–3.632)	.207	Random	81.9
AA vs AT + TT		0.640 (0.414–0.990)	.045	Fixed	0
AT vs AA + TT		1.366 (1.115–1.673)	.003	Random	84.7

Caucasian	1				
A vs T		0.735 (0.502–1.076)	.113		/
AT vs AA		1.897 (1.147–3.137)	.013		/
AT vs TT		1.842 (0.703–4.828)	.214		/
AA vs TT		0.971 (0.381–2.475)	.952		/
AA + AT vs TT		1.233 (0.495–3.073)	.653		/
AA vs AT + TT		0.588 (0.366–0.946)	.029		/
AT vs AA + TT		1.890 (1.156–3.091)	.011		/

Asian	2				
A vs T		1.701 (0.646–4.480)	.282	Random	89.1
AT vs AA		1.177 (0.393–3.527)	.771	Fixed	0
AT vs TT		1.892 (0.602–5.950)	.275	Random	90.9
AA vs TT		1.014 (0.346–2.978)	.979	Fixed	0
AA + AT vs TT		1.888 (0.598–5.962)	.278	Random	91.0
AA vs AT + TT		0.976 (0.332–2.867)	.965	Fixed	0
AT vs AA + TT		1.279 (1.023–1.598)	.031	Random	90.9

**Figure 2:**
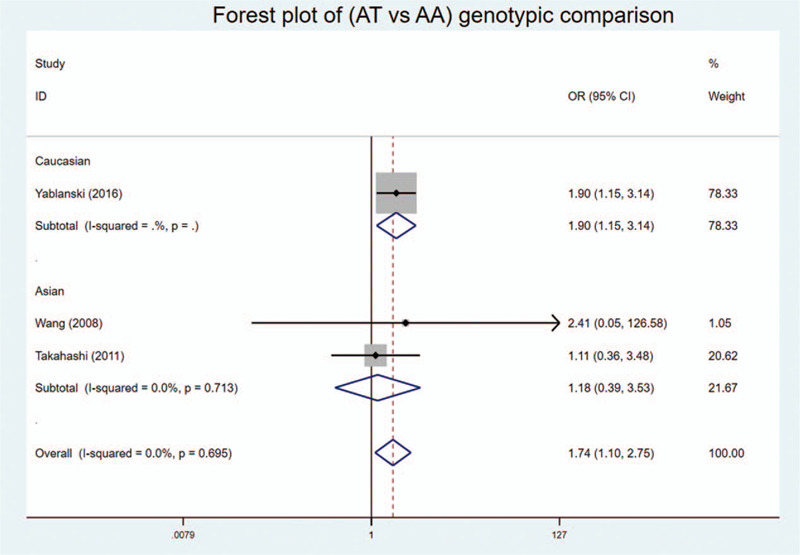
Forest plot of the codominant heterozygote model (AT vs AA) of genotypic comparisons between the rs10488682 polymorphisms and AIS: significance in overall and Caucasian, OR > 1.

**Figure 3:**
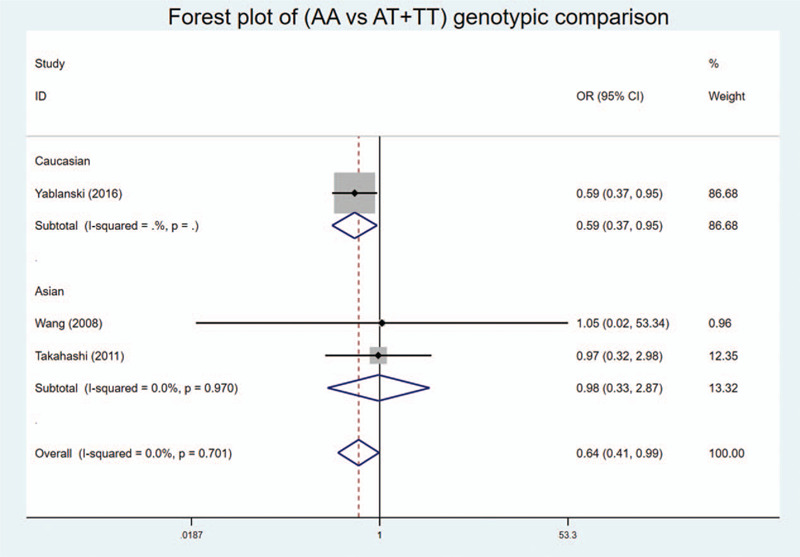
Forest plot of the recessive model (AA vs AT + TT) of genotypic comparisons between the rs10488682 polymorphisms and AIS: significance in overall and Caucasian, OR < 1.

**Figure 4:**
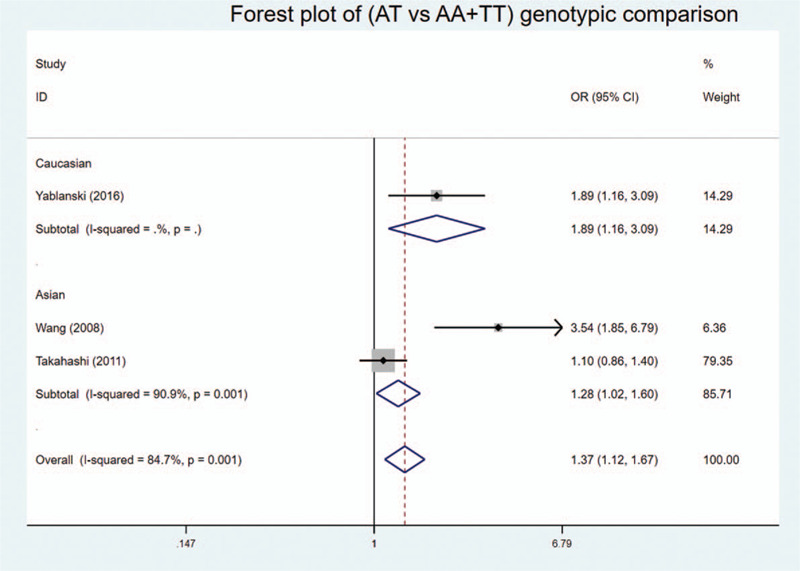
Forest plot of the over-dominant model (AT vs AA + TT) of genotypic comparisons between the rs10488682 polymorphisms and AIS: significance in overall and both ethnicities, OR > 1.

We found statistically significant associations in codominant heterozygote model, recessive model, and over-dominant model of genotypic comparison: (AT vs AA: OR = 1.741, 95%Cl = 1.100–2.753, *P* = .018 < .05, *I*^2^ = 0%) (Fig. 2); (AA vs AT + TT: OR = 0.640, 95%Cl = 0.414–0.990, *P* = .045 < .05, *I*^2^ = 0%) (Fig. 3); (AT vs AA + TT: OR = 1.366, 95%Cl = 1.115–1.673, *P* = .003 < .05, *I*^2^ = 84.7%) (Fig. 4).

Although without statistical significance, we found obvious OR > 1 in the other heterozygote model (AT vs TT: OR = 1.855) and dominant model (AA + AT vs TT: OR = 1.657) of genotypic comparison. No statistical significance was found in allelic comparison and homozygote model of genotypic comparison. Different models were used according to *I*^2^ in each comparison. Sensitivity analysis showed that the omit of Yablanski's study changed the summary OR and significance, which indicated the specific gene background of each ethnicity might modify the risk of AIS. It also showed that the omit of Takahashi's study changed the summary OR but not the significance, which is due to the large sample size of this study.

**Figure 5:**
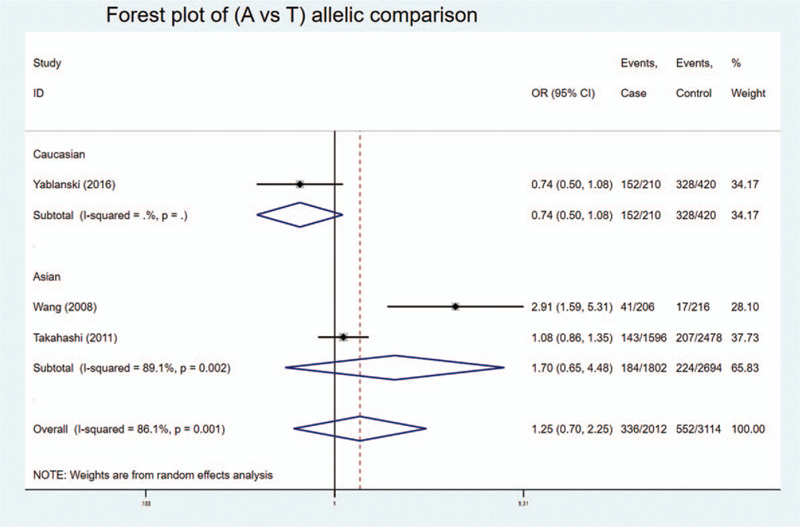
Forest plot of (A vs T) of allelic contrast comparison between the rs10488682 polymorphisms and AIS: no significance, OR varied by ethnicity.

In addition, subgroup analysis by ethnicity showed similar results. Statistical significance was found in heterozygote and recessive model in Caucasian: (AT vs AA: OR = 1.897, 95%Cl = 1.147–3.137, *P* = .013 < .05) (Fig. 2); (AA vs AT + TT: OR = 0.588, 95%Cl = 0.366–0.946, *P* = .029 < 0.05) (Fig. 3). And statistical significance was found in over-dominant model in both Caucasian and Asian: (AT vs AA + TT: OR = 1.890, 95%Cl = 1.156–3.091, *P* = .011 < .05) and (AT vs AA + TT: OR = 1.279, 95%Cl = 1.023–1.598, *P* = .031 < 0.05, *I*^2^ = 90.9%) (Fig. 4), respectively. No statistical significance was found in allelic comparison, where Caucasian had an obvious OR < 1 while Asian's OR > 1 (Fig. 5). This proved that ethnicity might be the source of between-study heterogeneity.

Subgroup analysis by gender was not performed, because only one of the studies targeted female, while others recruited both genders and did not report detailed genetic information.

## 4 Discussion

Researchers have been looking into the causes of idiopathic scoliosis for decades. However, the etiology and pathogenesis of AIS remained unsolved. Now scientists believe the pathogenesis of AIS includes multiple factors. Due to the positive family history of patients and the approval of twin studies, it is believed that gene plays an essential role in AIS.^[8]^ With the wide application of GWAS, researchers have obtained critical insights into the genetic etiology. However, due to the phenotypic and genetic heterogeneity, there are still obstacles in finding clear genetic associations.

Melatonin plays an important role in human biological function, such as circadian rhythm, puberty sexual maturation, aging, bone structure, and so on.^[22]^ It is primarily synthesized and secreted by the pineal gland and regulated by light and darkness, which is regarded as “chemical signal of night” and conveys information of circadian rhythms to body structures.^[29]^ Thus, melatonin mediates and a number of physiological activities, including sleep/wakefulness, immunity, cell proliferation, bone metabolism. The inactivity or deficiency of melatonin may lead to physiological disorders.

Since the experiment of pinealectomy-induced spinal curvature in chickens,^[23,24]^ huge amount of literature has reported various kinds of animal models to verify the relation between melatonin-deficiency and scoliosis, which advances our knowledge of the role of melatonin in bone metabolism.^[30]^ However, it is still not clear whether the positive results in animal models are applicable to human.

Although the direct mechanism of melatonin on scoliosis remains uncertain, one of the possible explanations is that during the sleep–waking cycle, the alteration of postural activity affects not only the limbs, but also the axial musculature. The reduce of melatonin and its precursors may suppress the posture and lead to hypotonia, which affects the axial muscle and develops scoliosis.^[31]^

Besides, researchers have also noticed the melatonin signaling pathway. A defect in melatonin metabolism could cause similar effect as melatonin-deficiency, which can also relate to the onset of scoliosis.^[25]^ Many genes of melatonin metabolism have already been investigated. TPH1 gene is one of them, and it encodes a crucial rate-limiting enzyme in the synthesis of serotonin in pineal cells. Serotonin is an intermediary of melatonin synthesis pathway, so reducing serotonin may reduce the synthesis of melatonin and cause AIS.^[21]^ Therefore, TPH1 has become a susceptible gene that mediates melatonin biosynthesis. The variation of it is possibly related to the predisposition of AIS.

The TPH1 gene encodes members of the aromatic amino acid hydroxylase family. It locates on chromosome 11q13. One of its SNP, rs10488682, is located at −173 of TPH1 promoter.^[32]^ This SNP may alter the promoter activity and affect the transcription of TPH1 gene. Then, the translation of the critical enzyme in serotonin synthesis could be affected. Therefore, it is linked with reducing melatonin and also the onset of disease.^[21]^

Our meta-analysis of 3 studies involved 1006 AIS cases and 1557 controls of Bulgarian, Chinese, and Japanese populations. It showed statistically significant results in heterozygote model (AT vs AA) and recessive model (AA vs AT + TT) in both overall and Caucasian populations, as well as over-dominant model (AT vs AA + TT) in both populations. Obvious OR > 1 with no significance was found in heterozygote model (AT vs TT) and dominant model (AA + AT vs TT). OR ≈ 1 was found in homozygote model (AA vs TT). Besides, OR values in allelic comparison (A vs T) vary by ethnicity (Caucasian OR < 1 and Asian OR > 1).

Usually, genetic studies of SNP yield results supporting a certain “risk allele” and a “dominant model effect” or “recessive model effect” of this allele. Such results are in accordance with a typical Mendel's law. However, in our meta-analysis, allele frequency seemed to vary in different ethnicities. Samples in Caucasian study of tend to have more AA genotype with very few TT genotype. Meanwhile, Asian hardly has any AA genotype and most of its samples are TT genotype. This suggested different gene distribution in each population. Ethnicity might be a vital modifier of the disease and a possible source of between-study heterogeneity. We could not simply consider either of the alleles in this SNP to be risk allele with regard to different OR values.

In addition, genotypic comparisons with all the models above did not fit Mendel's law. Because when a recessive model is found significant with OR < 1, it is hardly possible that a dominant model is nearly significant with OR > 1, and a homozygote model OR ≈ 1. We therefore conducted the over-dominant model to compare heterozygote with both homozygotes, and found positive results. Thus, it is reasonable to infer that both homozygous (AA & TT) genotypes are protective, while heterozygous AT genotype is risky. The significance of the genotypic comparisons above was mainly caused by AT heterozygote.

Such hereditary phenomenon is not rare to find in nature. The over-dominant model of heredity results in a specialty of heterozygote, which is also known as “heterosis advantage (or disadvantage).”^[33]^ Hypothesis assumes that in certain gene loci, the interaction of different alleles leads to different phenotypes from either homozygote. This could explain the different gene distribution in 2 populations. Each population mainly consists of one of the homozygous genotypes (AA in Caucasian and TT in Asian), rather than an even distribution, because the combination of 2 different homozygotes leads to a heterozygous offspring, which could be risky to AIS. Also, the over-dominant phenomenon was reported in many human etiology studies.^[34,35]^ We believe the same works for the associations between TPH1 and AIS.

There are many possibilities for our results. Previously, genetic disease studies were often overstated relevance by a prior investigation and then questioned by replication researches with different genetic backgrounds. The major difficulty faced by IS genetic studies is phenotypic and genetic heterogeneity.

Even though Wang got positive results in his study of Chinese in 2008, subsequent replication study of Japanese by Takahashi in 2010 rejected his conclusion with a much larger sample size. Considering the publication bias that the positive findings tend to be published rather than negative ones, the first genetic association study of TPH1 might be a coincidence. Compared with individual studies,^[21,27,28]^ our results expanded the statistical power, providing a better understanding of TPH1 gene polymorphism in AIS etiology. With current comprehensive evidence, TPH1 is a possible candidate gene for sequencing to be applied in clinical diagnosis.

### 4.1 Quality of literature

Reporting quality of literature is vital to meta-analysis results. Deviation from HWE was detected in Yablanski's study with a *P* value of .0052 < .05, which might indicate selection bias. We tried to contact the author to confirm the genetic distribution, because the allele frequency and the summary OR of comparisons in this study were obviously contrary to former studies, but received no reply. In addition, none of the studies assessed population stratification, maybe because of the small sample size. Also, genotyping errors might be further sources of bias. However, we evaluated the literature methodological quality using the modified NOS. According to the guidance of the scale, all studies were considered qualified (scored 6 of 9). This guaranteed sufficient effectiveness of the result.

### 4.2 Strengths and limitations

With increasing number of studies on melatonin in the pathogenesis of AIS, the crucial enzyme gene of melatonin biosynthesis pathway, TPH1, has been investigated in 2 major ethnicities. It is the first systematic review of current TPH1 genetic studies. Our results are relatively different from most of the previous genetic etiologic meta-analyses, where a certain risky allele of gene locus was suggested. So far as the evidence indicated that there might be a genotypic diversity in SNP rs10488682 between Asian and Caucasian population. The distribution of alleles proved that the polymorphism of this locus could not indicate any allele was pathogenic. Unlike previous systematic review, our results approved a risky genotype, in which we revealed a special role of heterozygote in disease susceptibility.

Meanwhile, some limitations are to be addressed about this review. First, the number of studies and their sample sizes are quite limited. At present, only 3 articles of genetic association and 1 article of genetic sensitivity to brace treatment of AIS have been published. Second, current evidence did not cover populations other than Asian and European Caucasian, which might decrease the generalizability of the outcome. Third, these articles did not report the detailed genetic data of each gender, so subgroup analysis by gender was not available. The possible difference between male and female in TPH1 gene is still unclear. Fourth, only one study featured Caucasian. More studies of Caucasian are encouraged to confirm the positive results in this meta-analysis. Last, the searching only yielded English and Chinese language literature in 6 databases. Potentially relevant studies in other languages might have been skipped.

## 5 Conclusions

The currently available evidence presented in our meta-analysis suggested statistically significant correlations between the TPH1 rs10488682 polymorphisms and AIS, where heterozygote model (AT vs AA) effect, recessive model (AA vs AT + TT) effect, and over-codominant (AT vs AA + TT) effect were found. Heterozygous AT genotype seems to be risky in overall populations and ethnicity could be a modifier of the disease association. Continued investigations with larger scales of samples and other populations are needed.

## Author contributions

JL and ZY contributed equally to this article, including literature searching, quality assessment, data extraction, statistical analysis, and result discussion. MY was in charge of the whole project and guaranteed the accuracy of meta-analysis results and conclusions. All authors read and approved the final manuscript and agreed to submit.

**Conceptualization:** Junyu Li, Zexi Yang.

**Data curation:** Junyu Li, Zexi Yang.

**Formal analysis:** Junyu Li, Zexi Yang.

**Methodology:** Junyu Li, Zexi Yang.

**Project administration:** Miao Yu.

**Supervision:** Miao Yu.

**Writing – original draft:** Junyu Li, Zexi Yang.

**Writing – review & editing:** Miao Yu.

## Supplementary Material

Supplemental Digital Content
