# Supplementary material for: Association study of single nucleotide polymorphism in tryptophan hydroxylase 1 gene with adolescent idiopathic scoliosis: A meta-analysis
Source: Medicine (Baltimore). 2021 Jan 22;100(3):e23733. doi: 10.1097/MD.0000000000023733 (PMC7837909; doi:10.1097/MD.0000000000023733)
Supplement: Supplemental Digital Content [file medi-100-e23733-s001.doc]

##
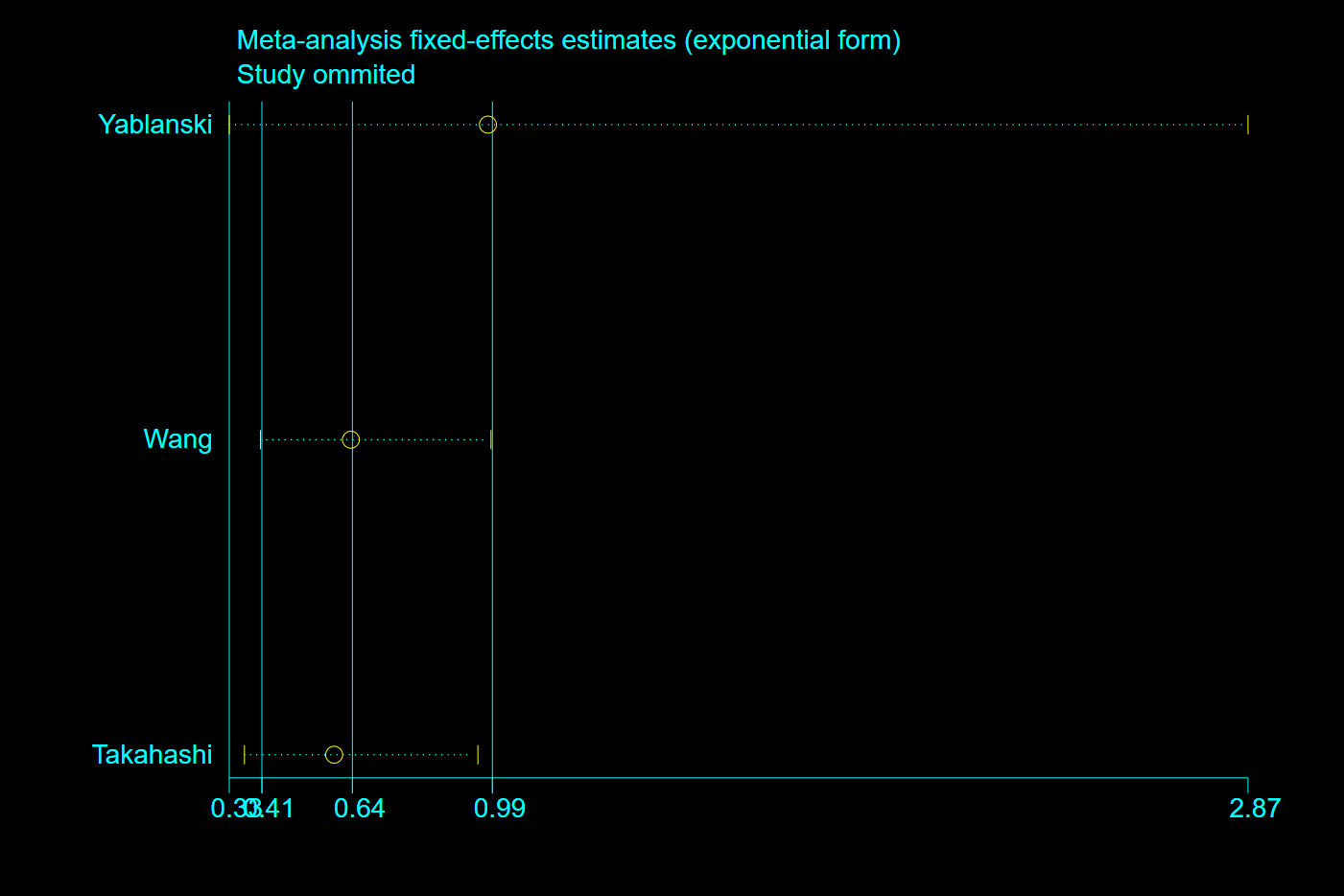


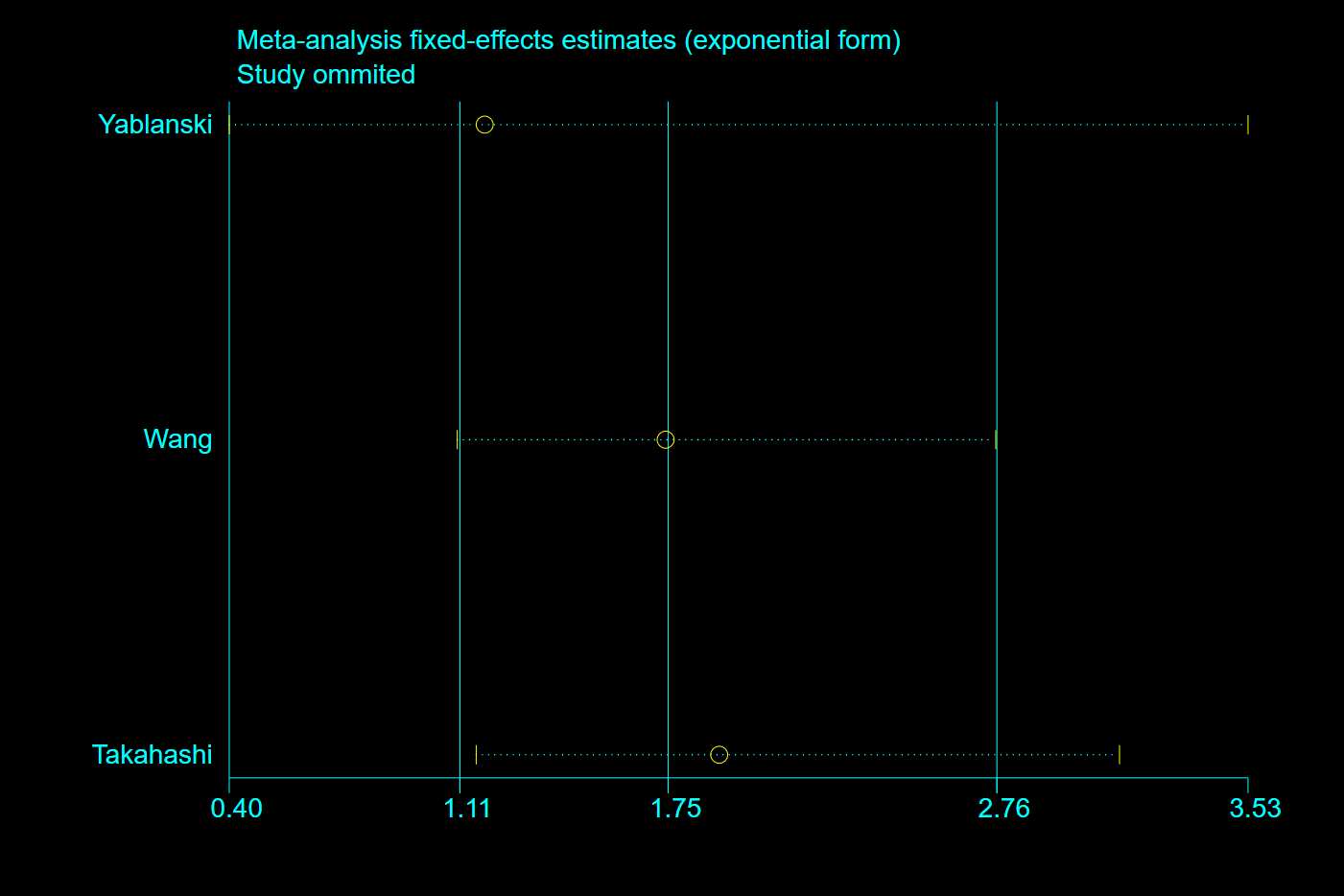


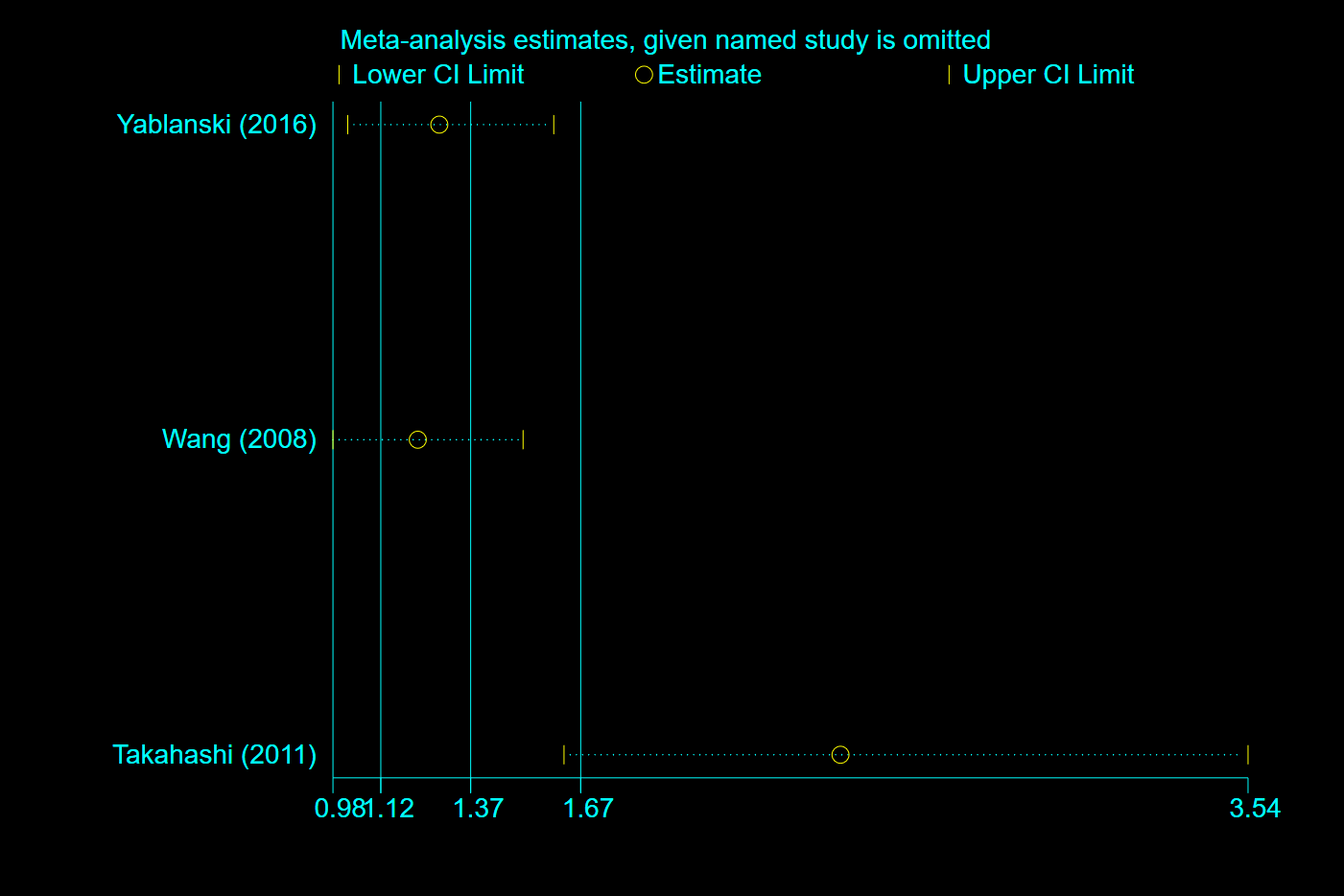


**Search Strategy**

According to the PRISMA guidelines, we managed to include all possibly relevant studies published until February 2020.

We systematically searched all the common databases, including: PubMed, Embase, Cochrane Library, Web of Science, Chinese Biomedical Literature and Wanfang database.

We especially featured in the genetic predisposition of AIS, with regard to the association between TPH1 gene and AIS disease. Thus, a series of search terms were used, as shown below:

‘TPH1’ OR ‘Tryptophan Hydroxylase 1’ is used to describe the TPH1 gene, together with its MESH term ‘TPH1 protein, human’ OR ‘Tryptophan Hydroxylase’ and Emtree term ‘tryptophan hydroxylase’ if applicable.

‘Adolescent idiopathic scoliosis’ OR ‘Idiopathic scoliosis’ OR ‘scoliosis’ OR ‘AIS’ OR ‘IS’ is used to describe the AIS disease, together with its MESH term ‘scoliosis’ and Emtree term ‘adolescent idiopathic scoliosis’ OR ‘idiopathic scoliosis’ OR ‘scoliosis’ if applicable.

Combining the two sums of search terms, we managed to obtain a literature search result as far as possible.

There was no language or publication status restriction. Additionally, we performed a manual screening of the published reference lists of relevant articles.
